# LONELINESS AND ACTIVITIES OF DAILY LIVING PERFORMANCE IN OLDER ADULTS: EXPLORING THE MODERATING ROLE OF COGNITION

**DOI:** 10.1093/geroni/igac059.2612

**Published:** 2022-12-20

**Authors:** Bianca Velez, Cynthia Dulaney

**Affiliations:** Xavier University, Cincinnati, Ohio, United States; Xavier University, Cincinnati, Ohio, United States

## Abstract

Resulting from changes in the sociodemographic landscape and the global pandemic, rates of loneliness have exponentially risen (Myers & Palmarini, 2017; Piette et al., 2020). Prior research demonstrates that loneliness is associated with an increased risk of cognitive and functional impairment (Cacioppo & Hawkley, 2009; de Jong Gierveld & van Tilburg, 1995; Holmén, et al., 1992; Wilson et al., 2007) as well as depression (Cacioppo et al., 2006; Kwon et al., 2017; Purtill, 2018). Although cognition has been studied as either a predictor or outcome of loneliness, it has yet to be examined as a moderator between loneliness and functional impairment (i.e., performance of basic and instrumental activities of daily living; ADLs, IADLs). Participants (N = 106) were community and non-community dwelling adults aged 65 years and older who completed four self-report measures assessing loneliness, ADLs/IADLs, cognitive functioning and depression. Hierarchical linear regression analyses were conducted to test the moderation model. Data revealed that cognition moderated the relationship between loneliness and IADLs. Follow-up simple slope analyses revealed that high cognition and low cognition were associated with decreased IADL performance amongst individuals with loneliness, and this association was stronger when cognition was low. The current study sheds light on the social, emotional, and physical disruptions the pandemic has caused in the lives of older adults. It highlights the need for interventions to enhance the quantity and quality of social relationships, and older adults’ involvement in physical and cognitive activities to uphold functional independence in later life.

